# Intraprosthetic Dislocation Following Reduction of Dual-mobility Total Hip Arthroplasty

**DOI:** 10.5811/cpcem.47211

**Published:** 2025-10-24

**Authors:** Matthias Barden, Marissa Benbassat, Emilio Benbassat

**Affiliations:** Eisenhower Health, Department of Emergency Medicine, Rancho Mirage, California

**Keywords:** hip dislocation, dual-mobility total hip arthroplasty, intraprosthetic dislocation, orthopedics, images in emergency medicine

## Abstract

**Case Presentation:**

A 62-year-old man was brought into the emergency department by ambulance with right hip pain and deformity following a suspected hip dislocation. A plain film radiograph confirmed the diagnosis. He was sedated for closed reduction at the bedside. Despite apparently successful reduction, his case was complicated by persistent intraprosthetic dislocation of a polyurethane liner component of his prosthetic joint articulation. Computed tomography confirmed displacement of the liner, which required operative intervention.

**Discussion:**

Intraprosthetic dislocation is a complication specific to dual-mobility hip prosthetics, characterized by displacement of the polyurethane liner unique to this type of device. This liner was designed to offer some benefits over other types of hip prosthetics, including improved biomechanics and lower risk of dislocation. The liner can become dislodged during a hip dislocation and remain displaced despite successful reduction of the metallic prosthetic components. Due to its radiolucency, diagnosis can be challenging on plain radiographs, often requiring advanced imaging. This case highlights the importance of recognizing this potential complication in patients with dual-mobility prostheses.

## CASE PRESENTATION

A 62-year-old male with history of avascular necrosis of the femoral head status-post total hip arthroplasty presented to the emergency department via ambulance with concern for left hip dislocation. His initial total hip prosthesis was performed nine years prior and was complicated by loosening of the acetabular cup component. He underwent revision a year or two later with placement of a dual-mobility prosthetic C-STIM ATM (DePuy Synthes, RaynHam, MA). The dual-mobility class of total hip prosthesis indicates the presence of a polyurethane liner positioned between the metallic femoral head and acetabular cup components.^1^, ^2^

**Image 1:**
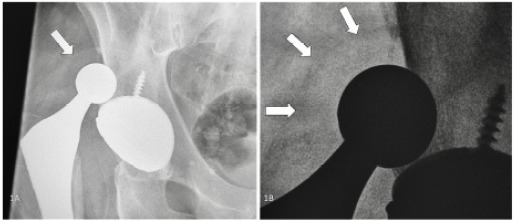
Radiograph demonstrating dislocation of a right dual-mobility hip prosthesis with a faint outline of the polyurethane liner displaced from the acetabular cup component, referred to as a “bubble” or “halo” sign (1A). The same radiograph, zoomed in and inverted to highlight the edge of the liner (1B).

He had done well after the revision until the day of the visit. He had been bent over gardening when he felt the joint become suddenly unstable and experienced immediate pain about the hip joint. Plain film radiography confirmed prosthetic hip dislocation. Noted retrospectively, this initial radiograph does reveal a subtle “bubble” or “halo” sign, suggestive of concomitant intraprosthetic liner dislodgement (Image 1).^3^

**Image 2:**
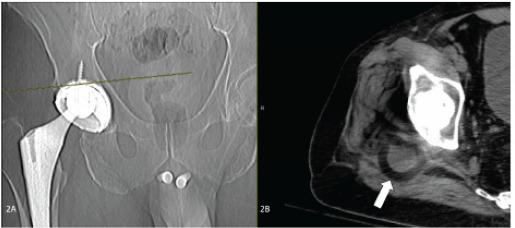
Computed tomography post reduction scout film (2A) and representative axial slice (2B) showing reduction of the femoral component into the acetabular cup, but with persistent intraprosthetic dislocation of the acetabular liner (arrow).

Following closed reduction under sedation, computed tomography confirmed reduction of the metallic portions of the prosthetic but also demonstrated dislodgement of the polyurethane liner component into the soft tissue about the hip, indicating intraprosthetic dislocation (Image 2).

The on-call orthopedic surgeon was contacted and took the patient to the operating room, where the acetabular liner was successfully replaced back into appropriate positioning within the prosthesis. The patient has done well since surgery without any further prosthesis complications on follow-up. He was advised to avoid further similar bending positions in the future.

## DISCUSSION

Dislocation remains a common major complication of total hip arthroplasty and a leading cause of revision surgery.^4^ Dual-mobility prostheses are designed with a polyurethane liner at the point of articulation between the femoral and acetabular metallic components. This design offers better joint mobility and lower rates of dislocation, particularly in high-risk patients.^5^ However, this design introduces the potential for intraprosthetic dislocation, a unique complication involving dislodgment of the polyurethane liner, which necessitates operative correction.^3^ While intraprosthetic dislocation was more prevalent in earlier dual-mobility designs, it remains a potential complication with contemporary prostheses and should be considered in any patient presenting with a dislocated dual-mobility total hip arthroplasty.^1^

*CPC-EM Capsule*

What do we already know about this clinical entity?

*Dual-mobility hip prostheses reduce dislocation rates but can develop intraprosthetic dislocation where the polyurethane liner becomes dislodged*.

What is the major impact of the image(s)?

*Images demonstrate the subtle ‘bubble’ sign on X-ray and CT confirmation of liner dislodgement, helping emergency physicians recognize this complication*.

How might this improve emergency medicine practice?

*Recognition of the ‘halo’ sign prompts CT imaging and early orthopedic consultation, preventing missed diagnosis and prosthetic component damage*.

The radiolucent polyurethane liner may manifest as a subtle “bubble” or “halo” sign on plain radiographs.^3^ Awareness of dual-mobility hip prosthetics and the potential for dislodgement of the intraprosthetic liner should prompt careful evaluation of plain films and consideration of axial imaging in cases of dislocation. Missed intraprosthetic dislocations will result in poor functional status, damage to metallic or ceramic components of the prosthesis, and higher risk for the need to replace the entire prosthesis.^2^ Open reduction or revision is likely to be required in cases of intraprosthetic dislocation; therefore, early orthopedic consultation should be considered.^3^
